# A versatile attention-based neural network for chemical perturbation analysis and its potential to aid surgical treatment: an experimental study

**DOI:** 10.1097/JS9.0000000000001781

**Published:** 2024-06-13

**Authors:** Zheqi Fan, Houming Zhao, Jingcheng Zhou, Dingchang Li, Yunlong Fan, Yiming Bi, Shuaifei Ji

**Affiliations:** a Department of Orthopaedics, The First Medical Centre, Chinese PLA General Hospital, Beijing; b Department of General Surgery, The First Medical Centre, Chinese PLA General Hospital, Beijing; c Senior Department of Otolaryngology-Head and Neck Surgery, The Sixth Medical Center, Chinese PLA General Hospital, Beijing; d Department of Urology, The Third Medical Center, Chinese PLA General Hospital, Beijing; e Department of Dermatology, The Seventh Medical Center, Chinese PLA General Hospital, Beijing; f Graduate School of PLA Medical College, Chinese PLA General Hospital, Beijing, People’s Republic of China

**Keywords:** chemical elements, deep learning, drug screening, surgical treatment

## Abstract

Deep learning models have emerged as rapid, accurate, and effective approaches for clinical decisions. Through a combination of drug screening and deep learning models, drugs that may benefit patients before and after surgery can be discovered to reduce the risk of complications or speed recovery. However, most existing drug prediction methods have high data requirements and lack interpretability, which has a limited role in adjuvant surgical treatment. To address these limitations, the authors propose the attention-based convolution transpositional interfusion network (ACTIN) for flexible and efficient drug discovery. ACTIN leverages the graph convolution and the transformer mechanism, utilizing drug and transcriptome data to assess the impact of chemical pharmacophores containing certain elements on gene expression. Remarkably, just with only 393 training instances, only one-tenth of the other models, ACTIN achieves state-of-the-art performance, demonstrating its effectiveness even with limited data. By incorporating chemical element embedding disparity and attention mechanism-based parameter analysis, it identifies the possible pharmacophore containing certain elements that could interfere with specific cell lines, which is particularly valuable for screening useful pharmacophores for new drugs tailored to adjuvant surgical treatment. To validate its reliability, the authors conducted comprehensive examinations by utilizing transcriptome data from the lung tissue of fatal COVID-19 patients as additional input for ACTIN, the authors generated novel lead chemicals that align with clinical evidence. In summary, ACTIN offers insights into the perturbation biases of elements within pharmacophore on gene expression, which holds the potential for guiding the development of new drugs that benefit surgical treatment.

## Introduction

Highlights

- Predictive models that utilize deep learning for drug screening can play an important auxiliary role in surgical treatment.
- Attention-based convolutional transpositional interfusion network (ACTIN) is not only a data-driven deep learning architecture, but also is with chemical prior information and good interpretability of predictions, which can generalize to a wider chemical space.
- ACTIN presents the notion of ‘chemical pharmacophore containing certain elements sensitivity differences in inter-cell lines’, which is great importance to personalized drug design and targeted treatments for diseases.
- ACTIN is with high reliability and can achieve the current SOTA only with a tenth of the usual trained sample size.
- ACTIN has great application prospects for screening drugs that benefit surgical treatment and comprehensive clinical decision.

The judicious application of perioperative drugs is pivotal for effectively reducing the incidence of infections, managing coagulation and hemostasis, and mitigating the occurrence of severe surgical complications, such as sepsis. However, traditional drug screening methods fail to meet the clinical demands. In addition, personalized drug treatment also faces great challenges due to the heterogeneity of patients. Therefore, optimizing the traditional drug screening methods, upgrading the drug screening technology, and overcoming the dilemma of personalized medicine are of great clinical significance to surgical treatment. Currently, drug repurposing and discovery fueled by artificial intelligence (AI) exhibit significant promise in expediting the new drug development cycle, elucidating the mechanisms of drug action, and forecasting preclinical drugs with potential for clinical translation^1^. Deep learning, as a method of AI, has exhibited the ability to uncover hidden correlations between drug targets and make reliable predictions^2–4^. For instance, DrugBAN, a deep bilinear attention network (BAN) framework, has shown a good predictive capacity for drug-protein interactions^5^. However, due to lacking information about key target proteins of most diseases, this method has a narrow application scope, especially for emergent epidemic diseases^6^. In contrast, gene expression profiles in disease states are readily available^7,8^. During disease conditions, alterations in cellular states could potentially impact numerous distinct genes; hence, gene expression profiles serve as extensively utilized informational tools for delineating disease. Using the potential link between drug and disease gene expression to predict personalized drugs can hold great promise. A compendium of chemical interferences in gene expression from ~80 cell lines, predominantly sourced from cancer and stem cells, library of integrated network-based cellular signatures (LINCS) L1000 dataset, has been employed for the prediction of drug-gene perturbations by cutting-edge techniques, like DeepCE (a mechanism-driven neural network-based method)^9^, chemical-induced gene expression ranking (CIGER) framework^10^, and multitask dose-dependent chemical phenomics model (MutliDCP)^11^. However, these models necessitate substantial training data, resulting in the limitation of clinical application. Besides, their interpretability and accuracy are still relatively lower.

Therefore, this work aims to develop an attention-based convolutional transpositional interfusion network (ACTIN), with minimal training data, to realize more authentic, high-throughput, and precise drug repurposing and discovery. We demonstrated that ACTIN has strong versatility and high accuracy, and it can dramatically improve the model performance in predicting chemical-induced gene expression and significantly outperform the current state-of-the-art models in drug repurposing. Much more importantly, in the ACTIN model, we also discovered perturbation bias of pharmacophore with certain elements on gene expression in different cell lines, which is of great significance to new drug development for improving perioperative treatment.

### The glossary

*Connectivity Map (CMap) and its extended project*. The CMap database contains gene expression profiles produced by cells treated with 1309 known functional compounds. In 2017, the second generation of CMap was built using the LINCS data^12,13^ with L1000 chip sequencing platform, expanding the database.

*Graph Neural Networks (GNNs) based model*. GNNs have been put forward as prospective graph-based methods^14^. GNNs can directly perform deep learning on graph-structured molecular data^3^. A key method from this domain is the Graph Convolutional Network (GCN), which directly targets molecular structural information^14^.

*Protein-protein interactions (PPIs)*. PPIs are the specific physical contacts established between two or more protein molecules due to biochemical events or electrostatic forces^15^. These interactions are involved in myriad biological processes, including intercellular communication, the regulation of metabolism and development, thereby playing a pivotal role in advancing biological research^16^. Presently, an increasing array of deep learning approaches are being applied to predict PPIs and their specific sites, facilitating a deeper understanding of underlying biological mechanisms^17^.

*Multilayer perceptron (MLP)*. It is a feed-forward neural network composed of multiple layers that contain several neurons^18^. The core principle of MLP is to process information through input, hidden, and output layers, using nonlinear activation functions and backpropagation algorithms to optimize the network. They are characterized by their simple structure and high versatility, making them suitable for various tasks^19^. MLPs offer benefits such as ease of implementation and adaptability, primarily being used in classification and regression machine learning tasks.

*Simplified molecular input line entry system (SMILES)*. SMILES is a notation method used to describe the structure of chemical molecules using a line of text. The principle behind SMILES is its ability to capture molecular connectivity and topology, which allows it to represent different chemical isomers distinctly^20^. This system is widely utilized in cheminformatics, to facilitate the rapid screening of compounds and modeling of molecular interactions.

*Principal Component Analysis (PCA)*. It is a statistical technique used to reduce the dimensionality of large datasets while preserving most of the original variance^21^. Principal components comprise a select set of linear combinations derived from the original variables, which collectively account for the maximal variance observed across the dataset. The principle behind PCA is to simplify the complexity in high-dimensional data while retaining the patterns and structures that are most important^22^.

*Attention heads (AH)*. AH is a fundamental component of the transformer architecture used in deep learning models. They function by focusing on different parts of the input data, allowing the model to selectively prioritize where to apply attention across the sequence for more effective processing. Each head computes attention scores, enabling it to capture various aspects and dependencies in the data.

*Feed-forward network (FFN)*. It is a type of artificial neural network. These networks are designated as feed-forward owing to their intrinsic architecture, which dictates information propagation exclusively in a unidirectional manner (forward)—namely, from the input nodes (units) directly toward the output units^23,24^. The primary principle behind feed-forward networks is the use of weighted connections, which are adjusted during training to minimize errors in the network’s predictions.

*Transformer mechanism*. The transformer mechanism is a type of neural network architecture widely used in natural language processing tasks. It relies solely on attention mechanisms to draw global dependencies between input and output sequences, avoiding the use of recurrent or convolutional layers^25^.

*Loss function*. A loss function is a mathematical function used to evaluate how well a model’s predictions fit the true data. The goal is to minimize the loss function, effectively minimizing the discrepancy between predicted and actual values. Common loss functions include mean squared error for regression tasks and cross-entropy loss for classification tasks in ACTIN we use smooth-L1 loss as a loss function^26^.

*TopKPooling*. TopKPooling is a pooling operation used in convolutional neural networks, particularly for tasks involving irregular data such as graphs or point clouds. It selects the k-highest values from the input feature map, preserving the locations of these values. This allows the network to focus on the most salient features while retaining their spatial information^27^.

*Node2vec*. Node2vec is a semi-supervised algorithm for learning continuous feature representations (embeddings) for nodes in a network or graph. It is inspired by the Word2vec algorithm used in natural language processing and aims to preserve the structural equivalence between nodes in the learned embeddings^28^.

## Method

**Figure 1:**
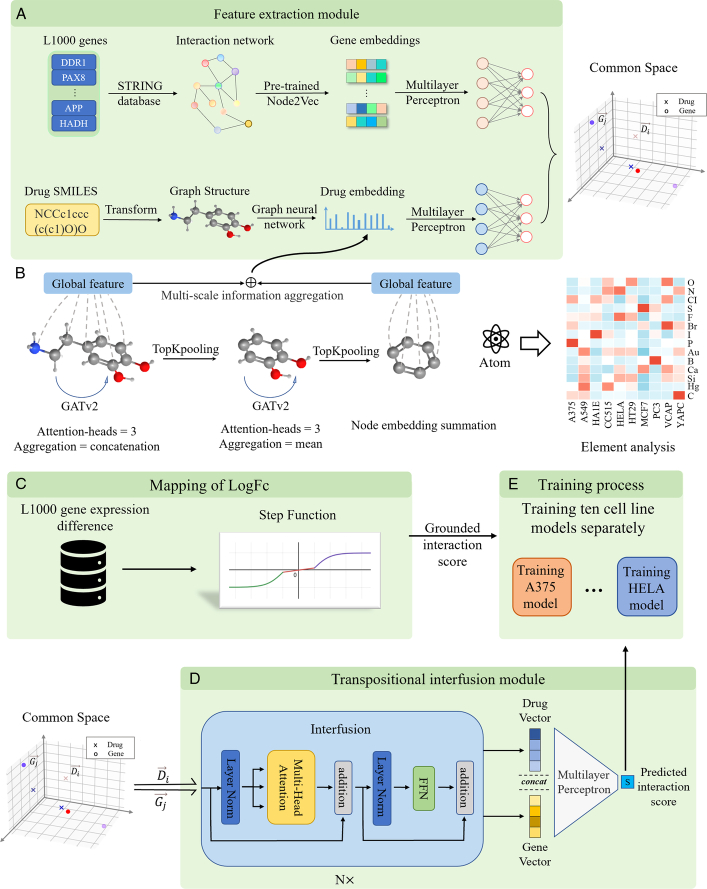
Overview of ACTIN. (A) Feature extraction module, transform genes and drugs into vectors and project them into common space. (B) Detail of extraction of drug features and subsequent element analysis using trained atom embedding. (C) The gene expression differences logFC are mapped to (-1,1) by step function. (D) Detail of transpositional interfusion module that simulates the interaction between drugs and genes. *N*=6, multihead=4. (E) Training 10 cell line models separately, and loss function is smooth-L1 loss.

To address the challenges associated with cell-line variations, we proposed a novel approach that split the L1000 dataset by cell line for separate training. This allowed us to treat each cell line as a separate dataset with its unique characteristics. To implement this idea, we developed the ACTIN model, which consists of three key components: (1) feature extraction component: This component transforms molecular and gene landmarks into vector representations $G_{j}$ and $D_{i}$ using GCNs (Fig. 1A and B). (2) Gene expression mapping component: this component projects the original log fold change (FC) values of gene expression into interaction scores between −1 and 1 (Fig. 1C). (3) Transpositional interfusion component: this component simulates the interaction between genes and molecules and predicts the interaction score $S_{i,j}$ between them (Fig. 1D).

### Extraction of drug molecular features

To capture drug molecular information, we employed a novel graph-based convolutional method called GATv2^29^, which utilizes an attention mechanism to calculate the relative importance of adjacent atomic nodes in molecular graph structures, enabling the capture of crucial elements or pharmacophores that impact medical effectiveness. We also used TopKPooling to prune graph structures, emphasizing the crucial roles of certain structures in molecular architecture. The detailed settings and illustrations are provided in Figure 1B.

For node X_i_, the calculation approach of X_i_’ is as follows after a graph-based convolution operation, N(i) represents all other nodes directly adjacent to X_i_. LeakyReLU is a useful activation function^30^. The attention coefficient α (_i, j_) is computed as follows:

**Figure:**
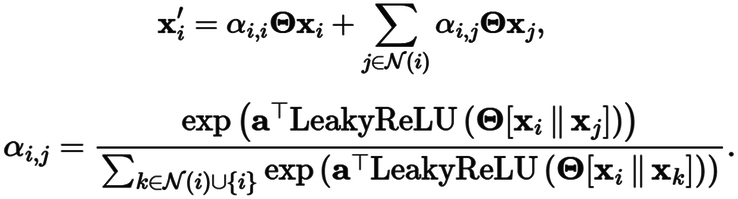


The graph information of molecular is extracted from SMILES, defining its atoms and chemical bonds as nodes and edges, molecules in the database mainly contain 14 types of atoms – ‘C, N, O, Cl, Ca, S, F, Br, I, P, Au, B, Si, Hg’. Each atom embedded in a one-hot vector is transformed into a 256-dimensional vector through an MLP. We use a series of GATv2-TopK pooling-GATv2-TopK pooling to obtain multiscale global features. Detailed settings and illustrations are provided in Figure 1B.

### Gene preprocessing

To obtain gene embeddings, we utilized the PPI network in the STRING database^31^ and pretrained Node2vec data which converts all 978 landmark genes in the L1000 database into 128-dimensional vectors^32^. MLP was used to map both the gene and drug vectors into a 256-dimensional common space. Subsequently, the Transpositional Interfusion Module was employed to identify potential associations between the given molecule and gene. The overall structure of the Features Extraction module is shown in Figure 1A.

### Mapping of gene expression

To comprehensively understand the biological meaning of logFC (log2 FC) values, we have designed a modified tanh(x) function called step-function (SF) to normalize gene expression differences. This function divided logFC scores into three distinct intervals, attenuating the influence of logFC values without substantial gene expression differences (−1 to 1) while highlighting upregulated gene expression (logFC >1) and downregulated gene expression (logFC <−1). This stepwise partitioning based on logFC values aimed to accentuate the gene expression differences that hold biological significance. The step-function formula is provided below, and the illustration is shown in Supplementary Fig S1a (Supplemental Digital Content 1, http://links.lww.com/JS9/C730).

**Figure:**
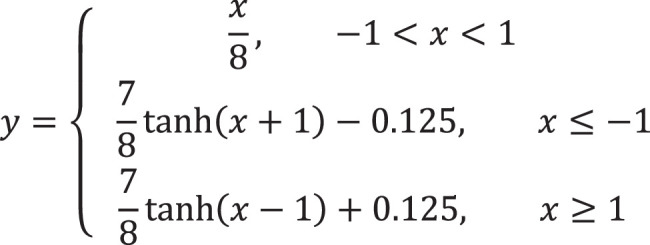


By implementing this method, we achieved a more refined and nuanced characterization of gene expression differences, enhancing the overall performance and interpretability of the model. This approach was tailored to capture the biological relevance of genes and mitigate the confounding effects of non-differential gene expression on the model’s efficacy.

### Transpositional interfusion module

Based on the data processing steps described above, the SMILES structure data of molecules and the landmark genes from the L1000 database were projected onto a common space of 256 dimensions. In this part, we designed a Transpositional Interfusion module to decipher the vector information of molecules and genes, aiming to explore the interaction relationships between molecules and genes. It is an enhanced encoder module based on the transformer architecture^32^. We used a prelayer norm to the input vector, previous studies have demonstrated that this modification^3,33^, as opposed to the original postlayer norm, leads to improved performance. In the subsequent layer, we employed a multihead attention mechanism to facilitate the transposition of drug and gene features^34^. Specifically, the attention mechanism transforms the input vectors (i.e. $G_{j}$ and $D_{i}$ ) into three distinct matrices: Q, K, V ∈ $R^{2\times d_{k}}$ , which are computed as follows:


$$Q=\;X\cdot \;W_{Q}$$

$$K=\;X\cdot \;W_{K}$$

$$V=\;X\cdot \;W_{V}$$

Here, $W_{Q}$ , $W_{k},W_{v}$ ∈ $R^{d\times d_{k}}$ represent the trainable parameter matrices, $d_{k}$ is the dimension of the transformed vectors, and X∈ $R^{2\times d}$ denotes a concatenation of $G_{j}$ and $D_{i}$ Subsequently, the attention-based representations for the input vectors are calculated as:


$$X\prime =\mathrm{softmax}(\frac{Q\cdot K^{T}}{\sqrt{d_{k}}})V$$

The resulting *X’* integrates information from both $G_{j}$ and $D_{i}$ , thereby enabling a mathematical simulation of the interaction between genes and molecules. In our work, we set $d_{k}$ to 64 and employed four AH, resulting in a multihead attention output with a shape of 2×4d_k_. By multiplying it with a trainable parameter matrix $W_{o}$ ∈ $R^{4d_{k}\times d}$ , we obtained $X_{o}$ , which has the same dimensions as X and captures information from all AH. After that, a short-cut addition was used to combine X and $X_{o}$ , followed by layer normalization, an FFN, and a final shortcut addition. These were the internal structures of interfusion (Fig. 1D).

After the multilayer interfusion process, a novel representation was obtained, which encompasses comprehensive information regarding molecular-gene interactions. Downstream prediction was accomplished by employing an MLP network.

### Training strategy

For training, we employed a new approach where chemical-gene pairs are associated, and a single gene with one chemical was imported to predict their interaction score. Each cell-line model was trained separately using the smooth L1 loss function (Fig. 1E). To optimize the training process, we employed mini-batch gradient descent with backpropagation. The hyperparameters of our architecture were kept the same across different cell lines to maintain the model’s universality. We selected the largest cell line dataset (MCF7) to determine the hyperparameters and fixed this structure for all cell line models. The dataset was divided into a 6:2:2 ratio for training, validation, and testing. For other cell line models, the dataset was divided into an 8:2 ratio for training and testing.

All pairs were shuffled and then sent into the training process (batch-size=1000). Each model was optimized using mini-batch gradient descent and updated parameters with backpropagation. Detailed model deployment and drug evaluation flow are described in Supplementary Fig. S1b and S1c (Supplemental Digital Content 1, http://links.lww.com/JS9/C730). By employing this approach, we aimed to overcome the challenges associated with cell-line variations and improve the performance and interpretability of our model.

### Experimental sample

To show the availability of our model in surgical conditions, we provided an appropriate example that can be followed by the average surgeon. For example, postoperative infection or sepsis seriously hinder the patient’s postoperative recovery. If the patient experienced a severe postoperative infection or sepsis that was difficult to treat with common antibiotics, we could input the transcriptome information of the patient’s biological samples into ACTIN to obtain potential FDA-approved compounds or drugs against postoperative infection or sepsis, quickly achieving personalized medication guidance.

## Result

### ACTIN model achieves state-of-the-art performance

**Figure 2:**
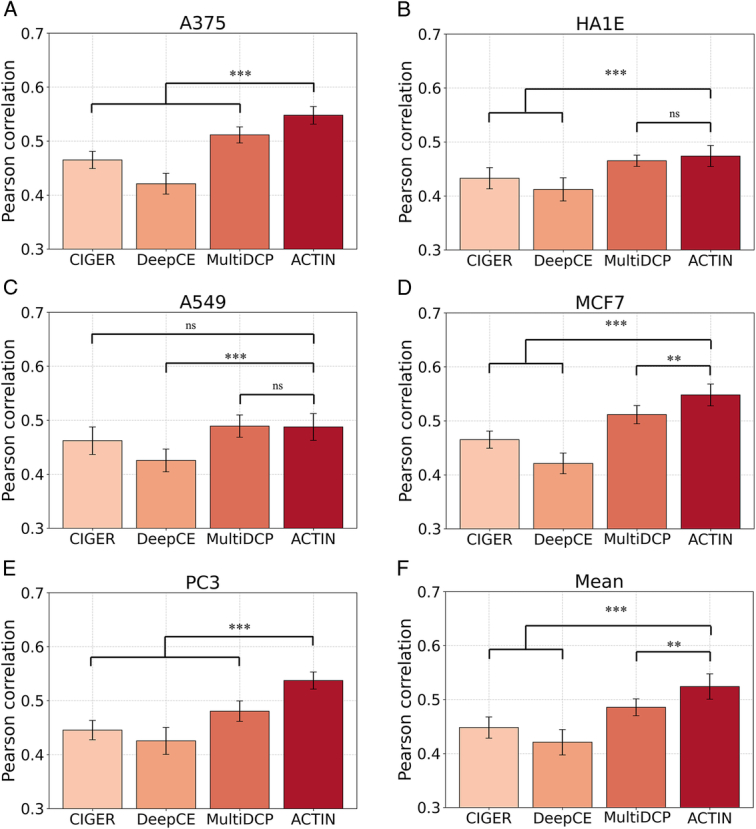
Pearson correlation of Model tests in different cell lines. (A) A375 (B) HA1E. (C) A549 (D) MCF7 (E) PC3 (F) Mean. The significance test used the fisher-Z test, **: *P*<0.01, ***: *P*<0.001. ns means no significance: *P*>0.05.

To exhibit the state-of-the-art predictive performance of ACTIN, we conducted the comparison between ACTIN and reported drug screening models, and the results were illustrated in Table 1 and Figure 2. Meanwhile, receiver operating characteristic (ROC)-area under the curve (AUC) and precision-recall (PR)-AUC were employed as quantitative metrics to realize binary classification performance comparison.

**Table 1:** The test accuracy comparison of ACTIN and other algorithms or drug prediction models of chemical gene perturbation.

		Down-regulation genes		Up-regulation genes	
Models	Sample size	ROC-AUC	PR-AUC	ROC-AUC	PR-AUC
ACTIN-VCAP	349	0.7623	0.8550	0.7191	0.5101
ACTIN-PC3	601	0.7721	0.8689	0.7612	0.5895
ACTIN-YAPC	140	0.7651	0.8378	0.7318	0.5464
ACTIN-MCF7	758	0.7939	0.8782	0.7758	0.6123
ACTIN-HT29	385	0.7536	0.8397	0.7195	0.5684
ACTIN-HELA	172	0.7306	0.8337	0.6782	0.4739
ACTIN-HCC515	299	0.7189	0.8269	0.7021	0.5416
ACTIN-HA1E	480	0.7646	0.8457	0.7423	0.5906
ACTIN-A549	285	0.7548	0.8881	0.7074	0.5024
ACTIN-A375	492	0.7963	0.8704	0.7687	0.6228
ACTIN-ALL^a^	3961	0.7684 ± 0.022	0.8596 ± 0.018	0.7419 ± 0.029	0.5727 ± 0.043
MLP	3961	0.5542	0.3391	0.5325	0.3463
CIGER	3961	0.7124	0.7951	0.6886	0.4819
DeepCE	3961	0.6955	0.7824	0.6821	0.4851
MultiDCP	3961	0.7072	0.7841	0.6973	0.4919

^a^ ACTIN-ALL is the weighted average of other ACTIN models.

In detail, ACTIN-MCF7 and ACTIN-A375 demonstrated superior predictive efficacy. Significantly, except for two cell lines with smaller samples (HCC515 and HELA), the accuracy of other eight cell line models surpassed that of other reported models, including MLP, CIGER, DeepCE, and MultiDCP. In the above eight models, the ROC-AUC and PR-AUC values for up-regulated genes increased by an average of 6.9 and 16.6%, while for down-regulated genes, the increases were 7.8 and 8.1%, respectively. In addition, several models with smaller sample sizes obtained a better prediction performance. For example, ACTIN-YAPC (ROC-AUC=0.7651, *n*=140) outperformed the ACTIN-HT29 (ROC-AUC=0.7536, *n*=385) (Table 1). Similar results were also observed in the comparison of ACTIN-A375 (ROC-AUC=0.7963, *n*=492) and ACTIN-PC3 (ROC-AUC=0.7721, *n*=601).

In addition, we used Pearson correlation to directly compare predicted and actual gene expression values (Fig. 2). We evaluated ACTIN models tested with the cell line data that was provided by CIGER, DeepCE, and MultiDCP testing sets (PC3, A549, A375, HA1E, MCF7). All models were evaluated using five-fold cross-validation (Fig. 2). ACTIN presented a better performance in A375, MCF7, and PC3 cell lines compared with existing models. On average, the Pearson correlation coefficient of ACTIN was significantly higher than other models, showing an improvement of 9.2%.

**Figure 3:**
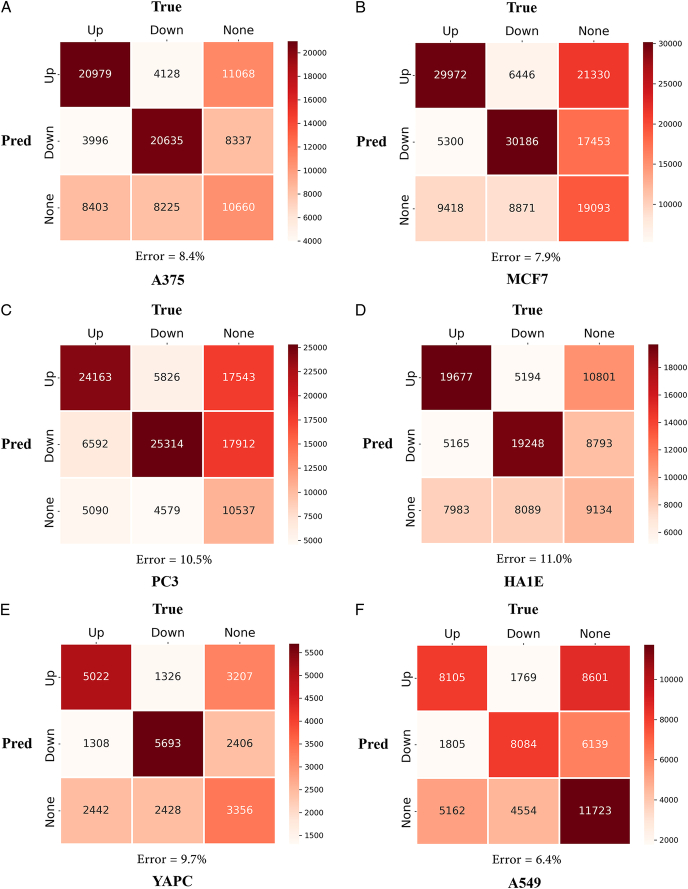
Confusion matrix of ACTIN. Numbers mean the test pairs. (A) A375 model. (B) MCF7 model. (C) PC3 model. (D) HA1E model. (E) HA1E model. (F) A549 model. Up: Up-regulation genes. Down: Down-regulation genes. None: Nonsignificant regulation genes (1>LogFC>-1). Error means the error rate of test samples, calculating the proportion of Prediction-Ture completely opposite samples. Another confusion matrix is shown in Supplementary (Supplemental Digital Content 1, http://links.lww.com/JS9/C730).

To further demonstrate the ability of ACTIN to identify up or down-regulated genes, a confusion matrix of all predictors (Fig. 3 and Supplementary Fig. S2, Supplemental Digital Content 1, http://links.lww.com/JS9/C730) and calculation of ROC and PRC (Supplementary Fig. S3, Supplemental Digital Content 1, http://links.lww.com/JS9/C730 and Supplementary S4, Supplemental Digital Content 1, http://links.lww.com/JS9/C730) were employed. The SF module involved three classification outcomes (note: if the prediction value was identical to the actual value, True labels, involving Up-Up or Down-Down, were given to classify samples, and then the remaining samples were considered as False, including Up-Down or Down-Up). The error prediction of above mentioned eight models accounted for 8.4, 7.9, 10.5, 11.0, 9.7, and 6.4% in A375, MCF7, PC3, HA1E, YAPC, and A549, respectively. Overall, for up-down or down-up gene pairs, the average error prediction accounts for only 9.47%, indicating ACTIN is with higher accuracy.

In summary, even with just 1/10 of the data from L1000 dataset, ACTIN-A375 model outperformed the latest gene-based drug-repurposing prediction models, even when a weighted average of the efficacy of all ACTIN-cell line models, the ACTIN-ALL model continued to demonstrate exceptional performance. These results underlined the consistent superiority of ACTIN in predicting drug-gene interactions.

### Disparity of pharmacophore containing certain elements

To further explore the interpretability of ACTIN, we conducted the parameters analysis. In the drug embedding module, all chemical elements were transformed into 256-dimensional vectors to investigate whether the element embeddings were consistent across different cell line models. For visualization purposes, we employed the PCA method to reduce the dimensions from 256 to 128 (retaining 97% of the variance). This section suggested that element differences in hub pharmacophore could interfere with cell line-specific effects tendency.

**Figure 4:**
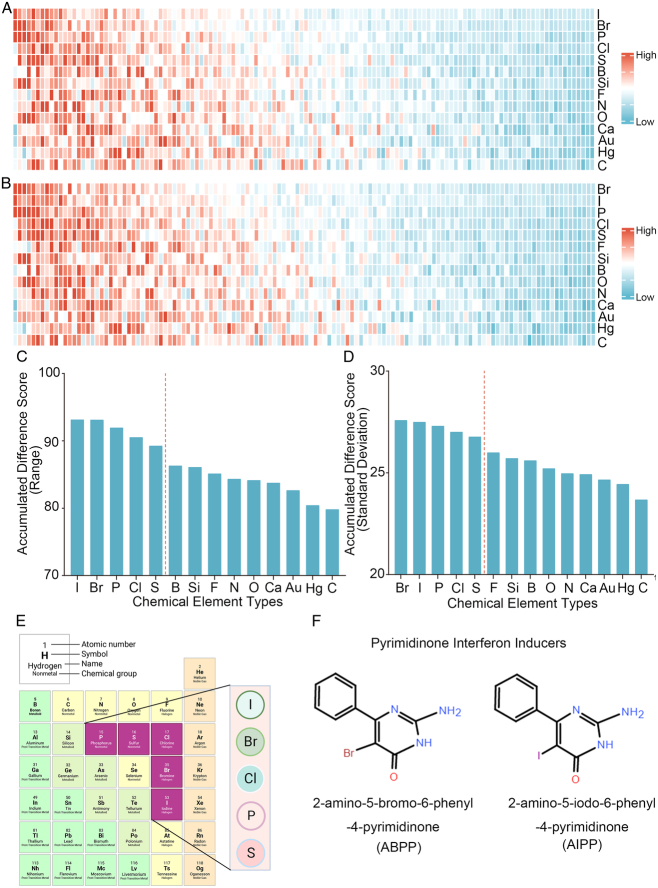
The range and SD of the 128-dimensional embeddings for 10 cell line models. (A–B) Heat map of elements embedding difference in range and SD of 128-dimension. (C-D) Sorted accumulative differences histogram of range and SD, and the red dashed line splits the top five elements. (E) The periodic table shows the proximity of these five elements. (F) Chemical structure of ABPP and AIPP.

In the hub pharmacophore and element identification results, the range and SD of the 128-dimensional embeddings were calculated to assess the difference in chemical element embeddings across the above models (Figs 4A–D). The range and SD heatmap presented that pharmacophores containing Br or I could exert a critical role in different cell lines which suggested that each cell line models should be trained separately to exclude the impact of pharmacophore element composition. Additionally, the variance in the element carbon (C) showed minimal effect across the above models, which was identical to the fact that C existed as a skeleton element in compounds (Fig. 4C and D). Meanwhile, the cumulative difference results revealed that the top five chemical elements with significant differences were iodine (I), bromine (Br), phosphorus (P), chlorine (Cl), and sulfur (S), which suggested pharmacophores involving S, P, I, and Br might have specific effect tendency in several cell lines. Remarkably, ACTIN successfully identified this element characteristic, despite not being provided with any prior chemical knowledge.

To explore the potential association between the above top five chemical elements, we found that these elements were situated adjacently in the periodic table (Fig. 4E). The results showed that element differences in hub pharmacophore could be distinguished by specific cell lines, which could explain that the antitumor properties of compounds presented diverse impacts on different cancer cell lines. For example, Br/I exhibited the largest variation, which indicated that their more variable effects among cell lines. Br has been verified to exhibit antitumor activity in carcinoma in situ (CIS) of the bladder and upper urinary tract^35^. Besides, both 2-amino-5-bromo-6-phenyl-4-pyrimidinone (ABPP) and 2-amino-5-iodo-6-phenyl-4-pyrimidinone (AIPP) were pyrimidine interferon inducers, and the only difference is Br and I (Fig. 4F). It was reported that ABPP was more effective than AIPP in inducing production of interferon when treating lung metastases of spontaneous fibrosarcoma, which may result from the chemical element difference^36^.

Our results demonstrated that ACTIN indeed acquired the knowledge of the interaction between functional groups containing particular elements and cell lines. Although our current analysis covered limited number of elements, the ability of ACTIN to handle small datasets allowed us to expand the chemical element difference heatmap continually.

### Attention mechanism identifies the dominant elements within pharmacophore in specific cell lines

After an overall analysis of the interaction between pharmacophores containing certain elements on cell lines, we focused on the distribution of chemical element importance within specific cell lines to investigate their role in drug-gene perturbations.

**Figure 5:**
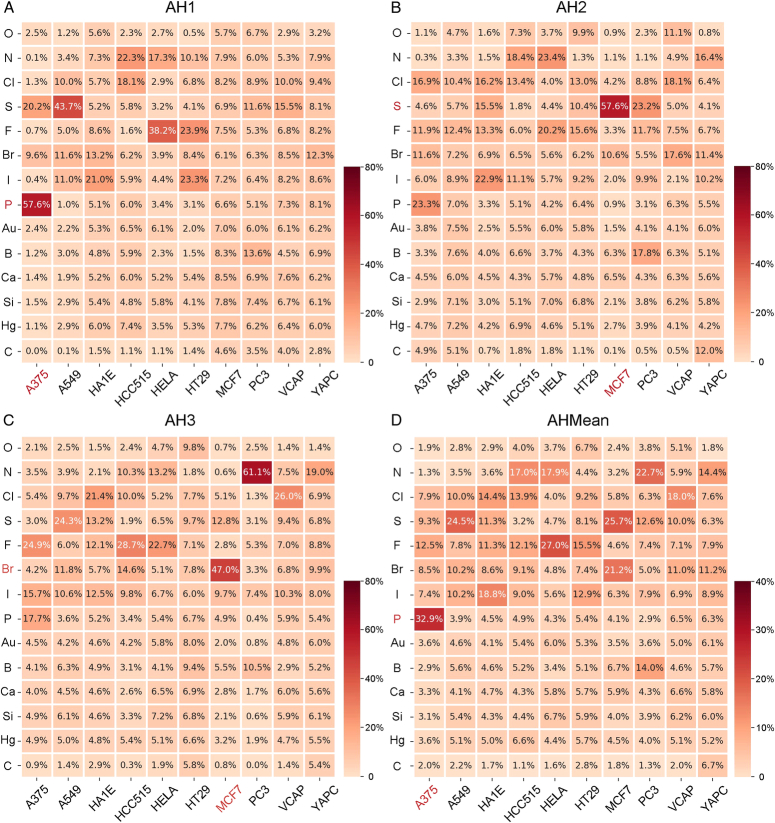
Heat map of element importance in each cell line model. (A-C) The quantitative importance of elements for different cell lines in attention-head 1, 2, 3. (D) The mean value of all attention heads.

Within the A375 cell line, it could be observed from attention heads 1 (AH1) that the weight proportion of P was 57.6% (Fig. 5A). This predominance is consistent across Attention Heads 2 and 3 (AH2 and AH3) highlighting the critical influence of the functional group containing P on gene expression in A375 cell line (Figs 5B and C). Similarly, in MCF7 cell line, the weight proportion of S was 57.6% in AH2, while Br made up 47% of the weight in AH3 (Fig. 5B and C). By examining the mean of AH, we could discern the significance of S and Br in creating differential gene expression when interfering with MCF7 cell line (Fig. 5D). These results suggested that functional groups enriched with S and Br may be the critical components determining the drug-induced gene expression differences in MCF7 cell line.

This cell line-specific analysis was of greater experimental significance, enabling us to concentrate on pharmacophores containing important chemical elements, which could trigger drug-gene perturbations. Furthermore, we could screen or design certain crucial pharmacophores with specific elements, which could be conducive to drug development and repurposing.

### Drug repurposing potential of ACTIN for treating fatal COVID-19 cases

To validate the practical capabilities of the ACTIN model, it was applied to drug screening for fatal COVID-19, resulting in the identification of potential candidate drugs for treating this critical condition. Firstly, differential gene expression profiles derived from lung tissue of a cohort of fatal COVID-19 patients (GSE180226) were analyzed. Subsequently, these differentially expressed genes were input into the ACTIN model, which facilitated the identification of 45 promising candidate drugs. After excluding the unknown ones, 35 drugs exhibited potential efficacy. Notably, 22 out of these candidates were underpinned by direct evidence, indicating their potential as effective treatments for fatal COVID-19, details of which were summarized in Supplementary Table 1 (Supplemental Digital Content 1, http://links.lww.com/JS9/C730). The action mechanisms of these drugs against fatal COVID-19 could be categorized into six domains:1) inhibition of the SARS-CoV-2 virus receptor angiotensin-converting enzyme 2 (ACE2), exemplified by Quisinostat, Loratadine, and Triciribine; 2) targeting SARS-CoV-2 replication, demonstrated by Lenvatinib and Honokiol; 3) restricting SARS-CoV-2 entry into cells, as with AZD5363, Agomelatine, and Cepharanthine; 4) blocking the SARS-CoV-2 main protease through drugs such as Parthenolide, Pyrrolidine-Dithiocarbamate, Sodium Tanshinone IIA Sulfonate, and Letermovir; 5) attenuation of inflammation, as with Rolipram, Tolimidone, Sacubitril, and Oseltamivir; 6) other mechanisms. The robust evidence supporting the efficacy of these drugs in treating COVID-19 served as an example to validate the accuracy and dependability of the ACTIN model in identifying potential treatments.

Furthermore, 13 additional drugs identified by the ACTIN model (Table 2) were not found to be used in COVID-19 treatment directly. However, their action mechanisms were closely associated with COVID-19 progression, indicating potential effectiveness. For instance, fludarabine and GSK650394 exhibited antiviral capabilities that could inhibit SARS-CoV-2 activity. NSAIDs have been advocated for analgesic or antipyretic treatment during COVID-19^47^, suggesting a potential indirect role for Ramifenazone in COVID-19 management. Histamine H2 receptor antagonists have shown potential in managing COVID-19-induced inflammation^38^. Therefore, Histamine H2 receptor antagonists like Roxatidine acetate and Nizatidine selected from the ACTIN model could be effective for fatal COVID-19 cases.

**Table 2:** The chemical information of potential drug candidates against fatal COVID-19 from ACTIN.

Drug	Mechanism of action	Known uses	Potential application	DrugBank ID	Structure
Fludarabine	DNA synthesis inhibitor	Cancers	Fludarabine has inhibitory activity on ZIKV, SFTS Phlebovirus, and Enterovirus A71	DB01073	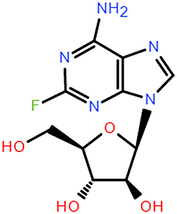
Ramifenazone	Non-steroidal anti-inflammatory drugs (NSAIDs)	Carrageenan edema and yeast fever	NSAIDs may play during SARS-CoV-2 infection and the development and progression of COVID-19^37^	NA	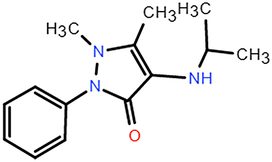
Roxatidine acetate	Histamine H_2_-receptor antagonist	Gastric and duodenal ulcers	H_2_ receptor antagonists in managing COVID-19 inflammation is evidenced^38,39^	DB08806	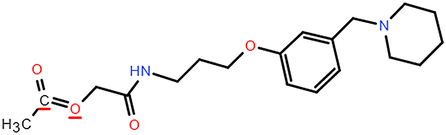
GSK650394	Serum and glucocorticoid-regulated kinase 1 inhibitor	Prostate cancer	Inhibit influenza virus replication	NA	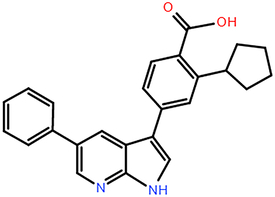
Mevastatin	HMG-CoA reductase inhibitor	Antitumor activity, cardiovascular diseases	Statins may be efficient SARS-CoV-2 main protease (Mpro) inhibitors^40^ Statin therapy is associated with decreased mortality in COVID-19^41^	DB06693	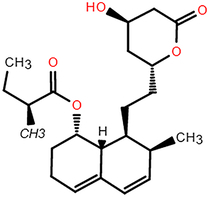
Lovastatin	HMG-CoA reductase inhibitor	Atherosclerosis and coronary heart disease	Statins may have a direct antiviral effect on SARS-CoV-2 by inhibiting its main protease^42^	DB00227	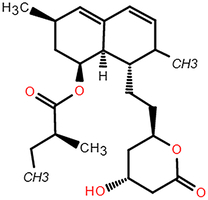
Nabumetone	COX-2 inhibitor	Anti-inflammatory	Possible inhibition of inflammation caused by SARS-CoV-2	DB00461	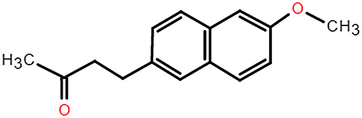
Nizatidine	Histamine H_2_-receptor antagonist	Duodenal ulcers	Similar to Roxatidine acetate	DB00585	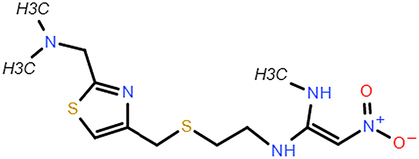
Bindarit	Monocyte chemotactic proteins inhibitor	Coronary Restenosis, Diabetic Nephropathy	Inhibiting NFκB-mediated inflammatory	DB12739	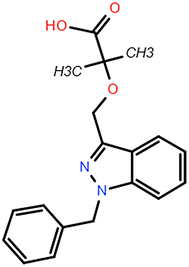
Halcinonide	Corticosteroid	Psoriasis	Corticosteroids might have the potential to reduce the risk of severe illness resulting from hyperinflammation in COVID-19^43^	DB06786	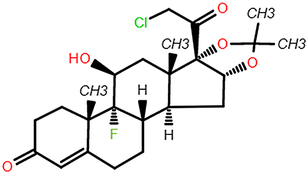
Beclomethasone	Corticosteroid	Prophylaxis of asthma attacks	The clinical trials of inhaled beclomethasone for COVID-19 were encouraged^44^	DB00394	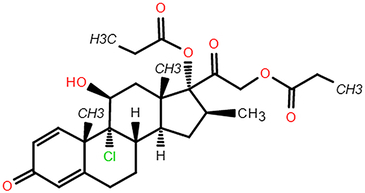
CDK1/5 inhibitor	Cyclin-dependent kinases inhibitor	Cancers	CDK may represent a novel drug target for SARS-CoV-2 treatment^45^	NA	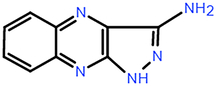
JANEX-1	Janus kinase 3 (JAK3) Inhibitor	Cancers	JAK inhibitors have antiviral and anti-inflammatory properties and represent a potential treatment for SARS-CoV-2 infection^46^	NA	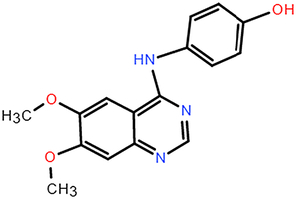

Additionally, experimental data suggested that statin drugs inhibited the SARS-CoV-2 main protease (Mpro) and blocked viral entry into host cells^48^. Clinical evidence indicated that statin therapy could reduce COVID-19 patient mortality, potentially qualifying Mevastatin and Lovastatin from the ACTIN model as candidates for severe COVID-19 treatment. The ACTIN model also identified anti-inflammatory and corticosteroid drugs like Halcinonide, Nabumetone, Bindarit, and Beclomethasone. Those drugs had been proven to control inflammation, which was considered as a key determinant in the severity of COVID-19 symptoms and patient mortality^49,50^. Extent studies have reported that cyclin-dependent kinases (CDK) and the JAK/STAT pathway might represent novel drug targets against SARS-CoV-2 infection^3,51^. In this context, ACTIN suggested that CDK1/5 inhibitors and JANEX-1 could potentially serve as novel therapeutic agents for severe COVID-19 cases.

These results suggested that the ACTIN model has demonstrated an impressive capacity for drug screening within the realm of COVID-19 therapeutics. The majority of the medications identified by ACTIN have been validated for their efficacy against COVID-19 through direct evidence. Additionally, the mechanisms of action of the remaining drugs were closely linked with the pathophysiology of COVID-19, but their actual efficacy requires further validation. Consequently, through empirical validation against COVID-19, the robust nature of our ACTIN model as a tool for drug screening has been demonstrated.

## Discussion

Perioperative drug use is crucial to improve surgical outcomes. The conventional drug development approach involves gaining an understanding of the disease’s pathophysiology and subsequently screening specific targeted drugs. Traditionally, drug screening involves systematically exploring potential chemicals for clinical translational value through laboratory experiments. However, such a method of drug development is hindered by a significant limitation: the extensive investment of human and material resources necessary for identifying and validating compounds for preclinical studies.

In this study, we proposed a novel deep learning framework (ACTIN) to predict gene expression profiles for de novo chemicals as well as utilize a phenotype-based drug-repurposing pipeline to identify potential treatments for diseases using existing drugs. This study introduced several advantages of ACTIN: 1) in the drug embedding stage, our study employed Graph GNN to handle the molecular structures of drugs. Our ACTIN model comprehensively considered the impact of drug molecular structure. 2) Our study constructed separate prediction models based on ten different cell line types. Compared to previous models that mix input from ten different cell lines without caring about cell line type differences^52–54^, the ACTIN model proposed in our study fully considered the heterogeneity of cell line types and achieved better predictive performance. 3) In the processing of the gene expression profile, by utilizing the PPI network from the STRING database to obtain vectors for 978 genes, our approach outperformed the simple one-hot encoding of the 978 genes as input to the deep learning network^52,54^. This method defined the distance between genes in advance, allowing the network to rapidly simulate interactions in a specific drug context. 4) The input stage of the ACTIN model accepted the paired input of drug vectors and gene vectors containing information on gene-gene interaction relationships. This approach addressed the issue of sparse training samples, and the simple input-output format facilitated network convergence. 5) Our study selected the GATv2 as the graph convolution method for drug feature extraction. Through attention coefficients (ranging from 0 to 1), the importance of chemical elements in the pharmacophore regarding the differential gene expression induced by the drug on cell lines was represented. 6) Notedly, the method in this study required only a few hundred data from L1000 chip sequencing to generate the model for any cell line, thus enabling targeted drug screening. These advantages make our model more general, and quick response to unknown diseases.

Furthermore, our model does not incorporate any specific input about the cell line information, where the term ‘cell line’ merely refers to just source of data generation. Therefore, our model framework does not necessitate sticking to cell lines, and organoids or other biological samples also can serve as data sources, which significantly broadens the applicability of our model, opening up new avenues for its utilization in diverse research domains and facilitating targeted drug screening in a wide range of contexts.

Meanwhile, previous drug prediction models have yet to consider the different effects of various chemical elements of the drug on gene expression differences in cell lines. Our findings suggested that: 1) the different pharmacophores containing special elements have different implications for different cell lines due to variations in their mathematical representation. 2) We have devised a respective elemental importance scale in different cell lines, which could facilitate targeted functional group design and drug development.

Our results also showed that, only by inputting the gene expression profiles under disease state, we can use trained models of human cell lines to rapidly predict likely effective drugs, which is particularly important in disease treatment. By applying the ACTIN model to analyze the differential gene expression profiles of lung tissue from fatal COVID-19 patients, we have identified 45 candidate drugs. Among them, 22 drugs have been supported by existing evidence as beneficial for alleviating symptoms or reducing mortality rates in COVID-19 cases. This validation reinforces the reliability of the ACTIN model. Additionally, 13 potential drugs exhibit molecular targets closely associated with the pathogenesis of COVID-19, suggesting their potential effectiveness in treating fatal cases. While further verification is required for these drugs’ specific effects in COVID-19 treatment, they hold promise as potential therapeutic options. Lastly, 10 drugs show potential benefits in the treatment of fatal COVID-19 cases, but limited information hinders a precise determination of their mechanisms of action. Collectively, the ACTIN model demonstrates exceptional accuracy and practicality in predicting therapeutic drugs for COVID-19 and presents a substantial breakthrough in the discovery of new drug candidates. Its application not only offers a broader selection of drugs for the treatment of fatal COVID-19 cases but also holds promising prospects in addressing future public health emergencies.

The innovation in the model architecture contributed to the success of ACTIN. First, in the feature extraction module, we used an attention-based GCN to automatically extract the structural features of drugs. Genes were also projected onto one vector space through the PPI network. Second, in the model training stage, we used a novel single drug-single gene training method that allows genes and drugs to be delivered in pairs, Third, we developed a new function to map gene expression values, enabling the network to pay more attention to their biological significance. The development of the ACTIN model marks a significant advancement in drug discovery, with its high-throughput precision screening capabilities and potential for clinical decisions. Although ACTIN is not directly involved in surgical procedures, it improves the efficiency and safety of surgical treatment by enhancing decision support and drug treatment strategies.

## Ethical approval

The data used in this study came primarily from public databases that have received ethical approval.

## Consent

No human subject is involved in this research.

## Source of funding

Not applicable.

## Author contribution

Z.F., S.J., and Y.B.: conceived the study; Z.F., J.Z., H.Z., and S.J.: collectively developed, coded, analyzed, and refined the method, as well as the paper; Z.F., Y.F., and Y.B.: contributed to algorithm development and method benchmarking; Z.F., J.Z., H.Z., and D.L.: provided a lot of advice on ACTIN construction. All authors wrote and read the paper, as well as approved the final paper.

## Conflicts of interest disclosure

The authors declare no competing interests.

## Research registration unique identifying number (UIN)

No human subject is involved in this research.

## Guarantor

The guarantor in this research is the corresponding author Shuaifei Ji.

## Data availability statement

The ACTIN’s codes, pretrained gene vectors and cell line data used in this work are available in the GitHub repository at https://github.com/ChinaFeinstein/ACTIN.

Availability of data and material: All data and needed to evaluate the conclusions in the paper are present in the paper and/or the Supplementary Materials, Supplemental Digital Content 1, http://links.lww.com/JS9/C730.

## Provenance and peer review

Not applicable.

## Supplementary Material

SUPPLEMENTARY MATERIAL
