# Supplementary material for: A versatile attention-based neural network for chemical perturbation analysis and its potential to aid surgical treatment: an experimental study
Source: Int J Surg. 2024 Jun 13;110(12):7671–86. doi: 10.1097/JS9.0000000000001781 (PMC11634177; doi:10.1097/JS9.0000000000001781)
Supplement: SUPPLEMENTARY MATERIAL [file js9-110-7671-s001.docx]

Supplementary Materials

# Methods

**Data acquisition**

The L1000 database mainly incorporates variations in gene expression profiles of 978 specific genes across 77 distinctive cell lines. These variations result from the application of 32,855 pre-identified drugs. This intricate process culminates in the creation of a comprehensive database delineating the disparate genetic expression instigated by the interaction of various drugs with these cell lines.

The current studies based on the L1000 database aim to identify potential candidate drugs with potential for clinical repurposing. This is achieved by establishing a correlation between the gene expression difference brought about by chemical-induced actions on cell lines and the discrepancies in gene expression observed amidst diverse disease states.

Although the L1000 database contains 77 cell lines, the sample size for most cell lines is insufficient to meet the data scale required for the construction of prediction models using deep learning. Consequently, precedent researches utilizing deep learning models for predictions based on the L1000 database have predominantly opted for the ten most common cell lines for model development, such as the DeepCE and CIGAR models. Similarly, based on the proportional differences of the 77 cell lines in the L1000 database and to reduce the noise of L1000, we selected the same top 10 cell lines for model construction, including A375 (human malignant melanoma cell line), A549, and HCC515 (human lung adenocarcinoma cell lines), HELA (human cervical cancer cell line), HT29 (human colorectal adenocarcinoma cell line), MCF7 (human breast cancer cell line), YAPC (human pancreatic adenocarcinoma cell line), VCAP and PC3 (human prostate cancer cell lines), as well as HA1E (human embryonic kidney cells). In total, this study included 3,961 instances of differential gene expression spectra for drug actions on cell lines.

The L1000 database is derived from the NIH's LINCS program. The original L1000 database can be accessed at https://www.ncbi.nlm.nih.gov/geo/query/acc.cgi?acc=GSE92742. The Bayesian-based LINCS L1000 dataset can be downloaded from <https://github.com/njpipeorgan/L1000-bayesian>.The transcriptomic data of fatal COVID-19 are obtained from GEO DataSets, and the GEO accessions is GSE180226.

**Model Deployment**

As ACTIN’s architecture was special, the deployment of the model needed to be emphasized. The procedure of ACTIN’s deployment is shown in Fig. S1. In the input stage, for the candidate drug, we ensure that the atoms present are among the 14 specific atom types mentioned earlier. Similarly, the gene input must belong to the L1000 dataset comprising 978 landmark genes.

Now for certain diseases, assuming that the differential expression genes (DEGs) of the disease have N genes inside 978 landmark genes and we have M candidate drug. To get the full gene profile (length N) for each drug, we do it step by step. Take one drug chemical SMILE paired with one gene, and send them into the trained model. Cycle N times to iterate all DEGs. So we get a drug’s predicted gene perturbation of length N. Loop M times to investigate all M candidate drugs. But how to choose one from M? Here, we have designed an Euler Distance-based Score function (EDS) function for evaluation.

**Drug evaluation using EDS**

By referring to the principles of drug screening in the CMap, the therapeutic efficacy of a drug for a specific disease depends on whether the gene expression differences induced by the drug perturbation exhibit an opposite trend to the gene expression differences under the disease state. For example, if a drug can downregulate the expression of a pathogenicity gene, it may possess therapeutic potential for that particular disease.

ACTIN model takes M×N drug-gene pairs as input, allowing us to obtain predicted results of drug-induced gene expression differences, with a length of N for each drug. These predicted results are matched against the gene expression differences under disease states, also of length N. For example, if N=400, we first calculate the proportion of genes exhibiting opposite trends in gene expression changes (i.e., upregulation and downregulation) and classify them under the "Acc" (Accuracy) category, representing genes with therapeutic significance. Conversely, the proportion of genes exhibiting the same trend in expression changes is categorized as "Err" (Error), representing genes with exacerbation significance. For instance, if among the 400 genes, a drug can significantly perturb 300 genes, with 200 having therapeutic significance and 100 having exacerbation significance, the drug's Acc would be 50%, and Err would be 25%. And the EDS can be computed using the following formula:

$$EDS=\sqrt{{(Acc-P)}^{2}+{(Err-P)}^{2}}$$

$$P=\frac{Acc}{1+Acc-Err}$$

Although the formula may seem like a bluff, the meaning of this formula is just a simple geometric length(Fig S1b). The significance behind this scoring design lies in our intention to prioritize Acc for candidate drug while imposing severe penalties for Err. In other words, to achieve a high score, a drug must exhibit a higher Acc to compensate for any increase in Err. This geometric-based scoring design elegantly reflects this fundamental principle. Hence, we employ the EDS for evaluating drugs. The users have the freedom to determine how to effectively utilize the 10 cell line models. They can opt to employ a subset of the models or perform a simple average of the drug prediction scores from the 10 models. This flexible approach empowers our model to adapt to diverse scenarios.

**Speed**

The primary advantage of this approach lies in its ability to simplify the experimental screening process, enabling high-throughput potential drug screening through model predictions. Through rigorous experimental validation, ACTIN has demonstrated its remarkable ability to handle high-throughput data. Its processing speed is approximately 234.3μs / (per drug × number of differential genes). When predicting all 978 landmark genes, the drug or compound screening model constructed in this study can efficiently screen over 10,000 molecules within 30 minutes (RTX 4080, 16GB).


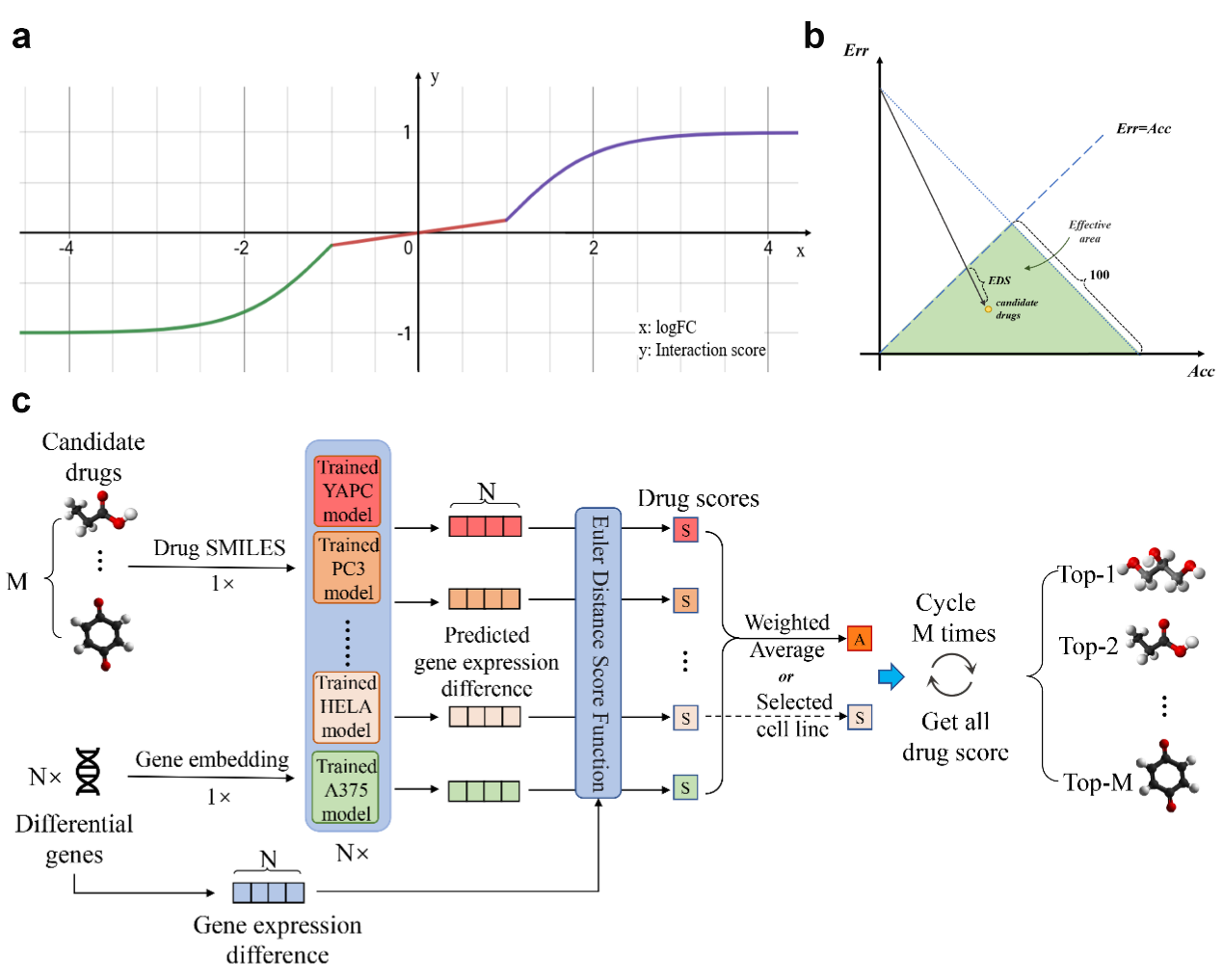


**Supplementary Figure 1.** The step function and deployment flowchart of ACTIN. **a.** The graph of step function of gene expression values (logFC) and interaction score. **b.** Deployment flowchart of ACTIN. Initially, one gene and one candidate drug are input into our cell-line models. Through repeated iterations, the models predict the resulting gene expression for each input combination, yielding a set of predicted gene expression differences for N differential genes. These predicted gene expression differences are then utilized, along with disease gene expression differences of length N, to compute drug scores using the EDS function. The iterative process is performed M times to obtain drug scores for all M candidate drugs. **c.** Geometric interpretation of EDS.

**
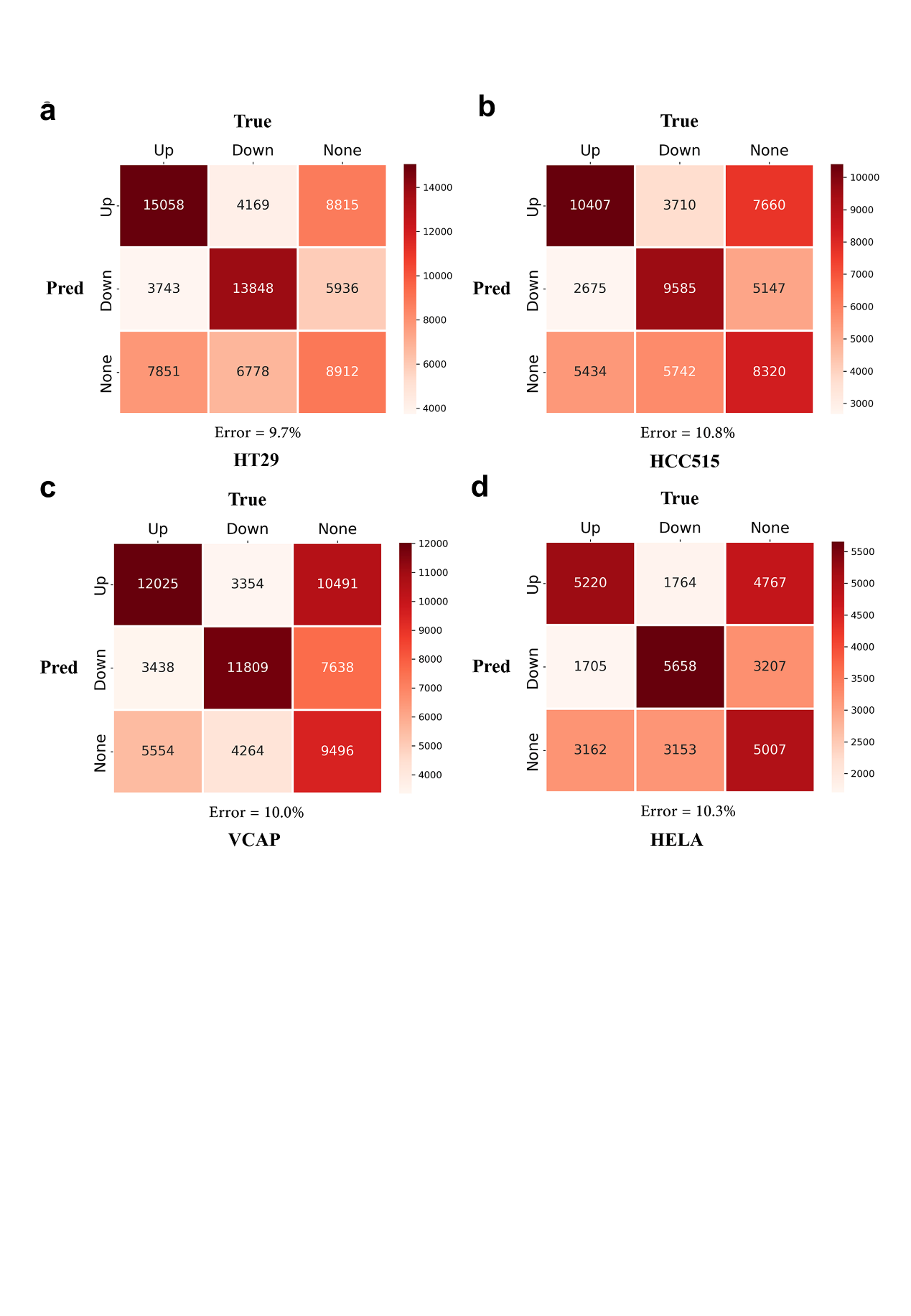
**

**Supplementary Figure 2. Confusion matrix of ACTIN**. **a**. YAPC model. **b**. HCC515 model. **c**.VCAP model. **d**. HELA model.

**
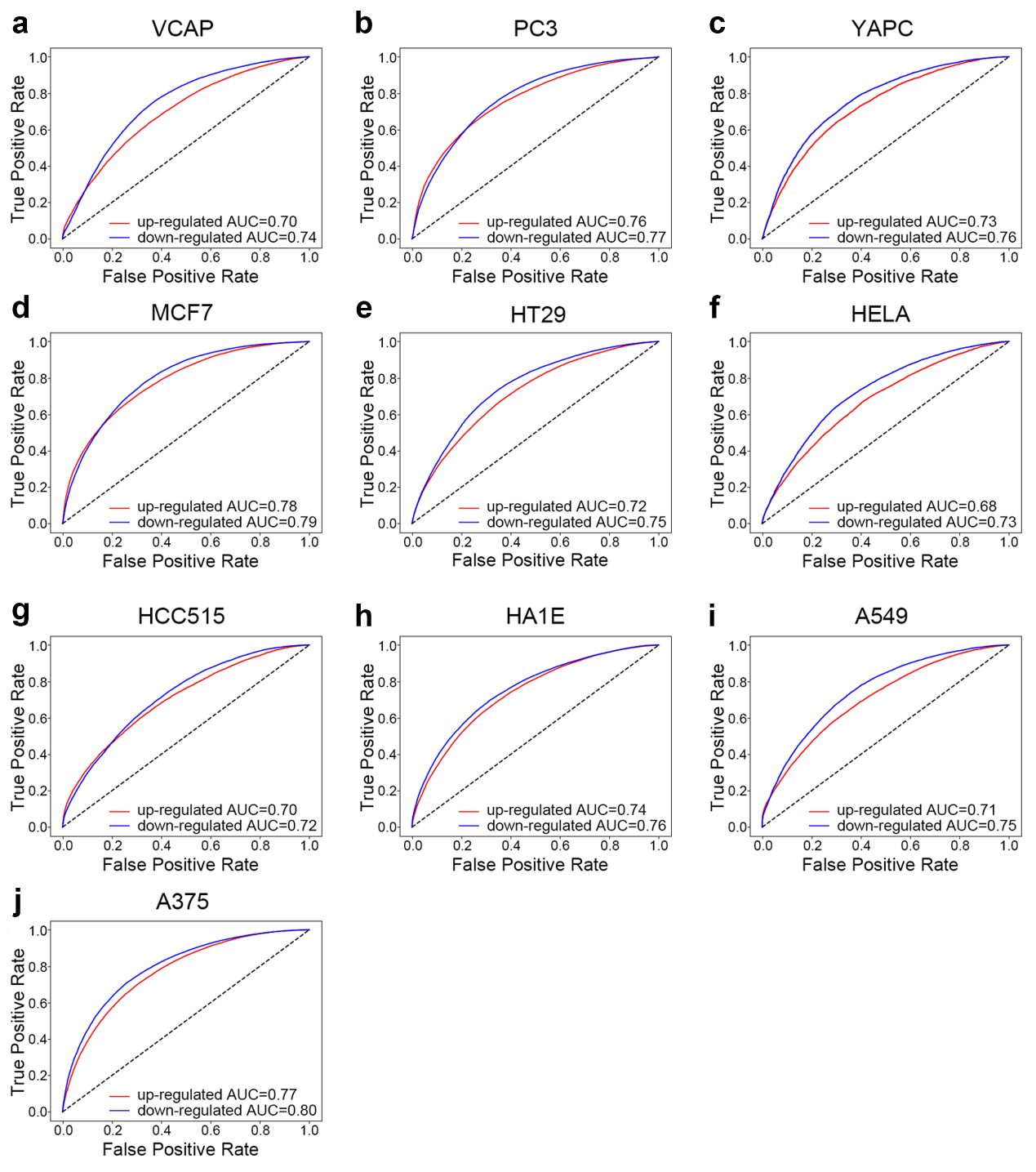
**

**Supplementary Figure 3. The ROC of all ACTIN cell line models**. **a-j.** Different cell line model performance.

**
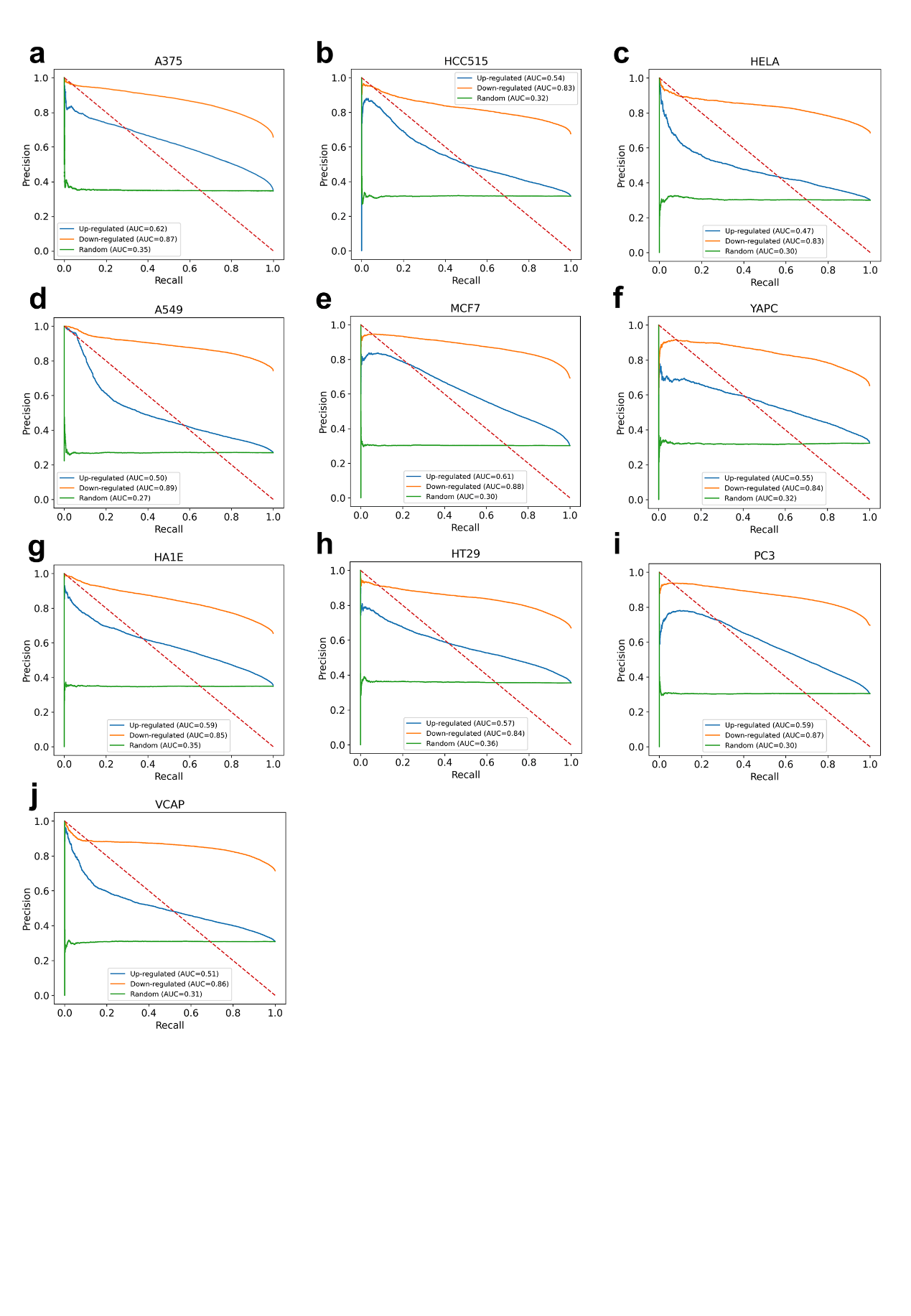
**

**Supplementary Figure 4. The PRC of all ACTIN cell line models**. **a-j.** Different cell line model performance.

Supplementary Table 1. The chemical information of identified potential drugs against COVID-19 from ACTIN.

| **Drug** | **Mechanism of action** | **Known uses** | **Evidences in COVID-19 treatment** | **DrugBank ID** | **Structure** |
| --- | --- | --- | --- | --- | --- |
| Tolimidone | Lyn kinase agonist | Type 2 diabetes | Reducing the occurrence and severity of lung symptoms from COVID-19 | DB16841 | 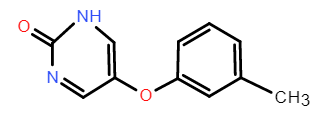 |
| Otamixaban | fXa inhibitor | Myocardial infarction | Inhibiting the protease activity of TMPRSS2 and block the entry of SARS-CoV-2 cell to ACE2-expressing cells(1, 2) | DB06635 | 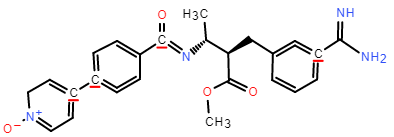 |
| Lenvatinib | Multi-targeted tyrosine kinase inhibitor | Hepatocellular carcinoma | Targeting SARS-CoV-2 replication subsequent to host-cell entry(3) | DB09078 | 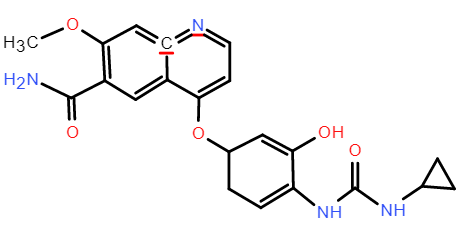 |
| Podophyllotoxin | Microtubule assembly and DNA [topoisomerase](https://www.medchemexpress.cn/Targets/Topoisomerase.html) inhibitor | Cancers | In Silico molecular docking revealed that podophyllotoxin exhibited good binding affinity to SARS-COV-2 proteins(4, 5) | DB01179 | 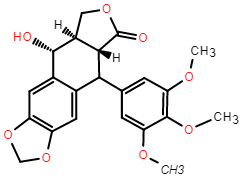 |
| Rolipram | Selective phosphodiesterases [PDE4](https://www.medchemexpress.cn/Targets/Phosphodiesterase%20(PDE)/pde4.html) inhibitor | Anti-inflammatory and antidepressant | Rolipram countered SARS-CoV-2 infection-caused airway inflammation by reducing [Cl-]i(6) | DB01954 | 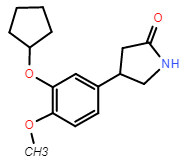 |
| Triciribine | DNA synthesis inhibitor,  [Akt](https://www.medchemexpress.cn/Targets/Akt.html) and HIV-1/2 inhibitor | Cancers,  HIV | Triciribine inhibited HMGB1-mediated inflammatory response in COVID-19, suppressed ACE2 expression and restricted COVID-19 infections;(7) | DB12405 | 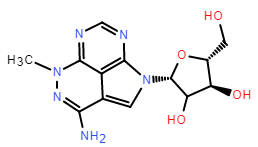 |
| Letermovir | Cytomegalovirus inhibitor | Kidney transplant  Stem cell transplant | Targeting the SARS-CoV-2 main protease (Mpro) and the key players of cytokine storm, TNF-α, IL-6, and IL-1β(8) | DB12070 | 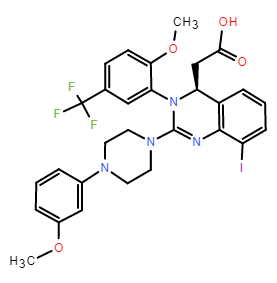 |
| Sodium tanshinone IIA sulfonate | Active lipophilic constitute of Danshen | Anti-oxidative and Anti-inflammatory,  Cardiovascular diseases | Direct interaction with COVID-19 drug target Papain-like protease (PLpro)(9) | NA | 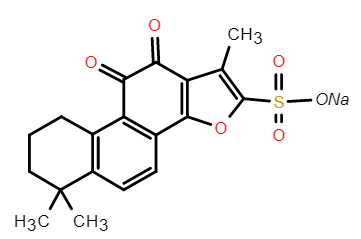 |
| Sacubitril | Neprilysin inhibitor | Heart Failure, Hypertension | Blocking NEP/angiotensin receptor type 1 (AT1Rs) required for ACE2 endocytosis in SARS-CoV-2 infection, and reducing inflammation related to COVID-19(10) | DB09292 | 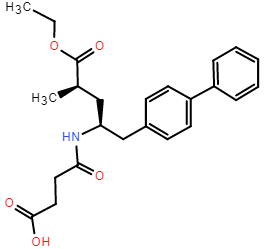 |
| Pyrrolidine-Dithiocarbamate | Nuclear factor κB (NF-κB) inhibitor | Anti-oxidative and anti-inflammatory | Alleviating SARS-CoV-2 nucleocapsid protein (N-protein)-caused acute lung injury(11) | NA | 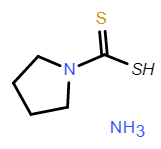 |
| Cepharanthine | Natural biscoclaurine alkaloid | Suppressing viral proliferation | Cepharanthine inhibited SARS-CoV-2 entry through the blocking of viral binding to target cells(12, 13) | DB16824 | 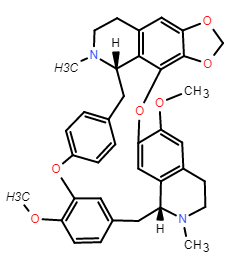 |
| Bexarotene | Retinoid X receptors (RXR) agonist | Cancers | Bexarotene exhibited potent in vitro anti-SARS-CoV-2 activity(14) | DB00307 | 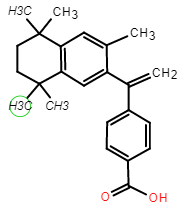 |
| Quisinostat | pan-[HDAC](https://www.medchemexpress.cn/Targets/HDAC.html) inhibitor | Cancers | Repositionable drug candidates for stabilizing the closed (substrate/inhibitor-bound) conformation of ACE2(15) | DB12985 | 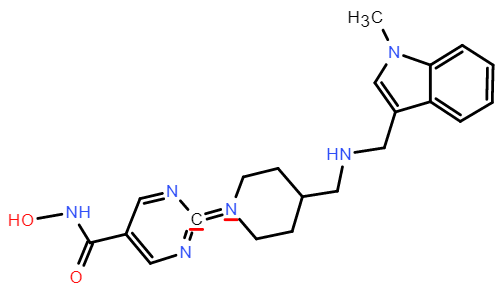 |
| Loratadine | Histamine H1 antagonist | Anti-dengue-virus activity and anti-inflammatory | Loratadine has inhibitory effect on SARS-CoV-2 spike pseudotyped virus viropexis by blocking spike protein-ACE2 interaction(16) | DB00455 | 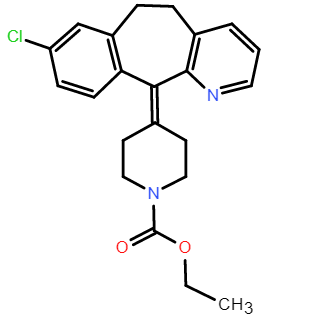 |
| Agomelatine | Specific agonist of [MT1](https://www.medchemexpress.cn/Targets/Melatonin%20Receptor/mt1.html) and [MT2](https://www.medchemexpress.cn/Targets/Melatonin%20Receptor/mt2.html) receptors | Depression | Agomelatine strongly prevent viral entry into the host cells through binding with SARS-CoV-2 receptor binding site and ACE2(17) | DB06594 | 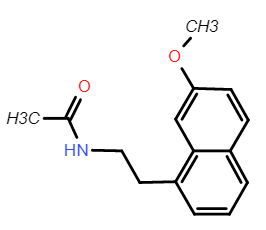 |
| Parthenolide | Sesquiterpene lactone | Cancers  Anti-inflammatory | Parthenolide exhibited good good binding with coronavirus papain-like protease (PLpro) and inhibited activity by allosteric regulation;(18, 19)  Parthenolide reduced hyperinflammatory states or cytokine storm in COVID-19(20) | DB13063 | 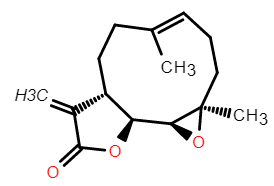 |
| Oseltamivir | Inhibitor of neuramidases of both influenza A (the most common type) and influenza B viruses | Flu virus | Oseltamivir shortened the median time to recovery of COV-D-19 patients(21) | DB00198 | 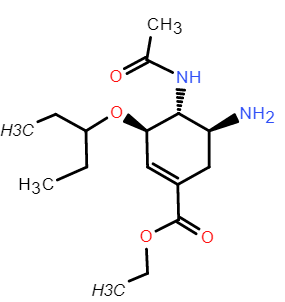 |
| AZD5363 | pan-AKT kinase inhibitor | Metastatic breast cancer | AZD5363 restricted the entry of SARS-CoV-2 into cells under non-cytotoxic concentrations via targeting PI3K/AKT signal pathway(22) | DB12218 | 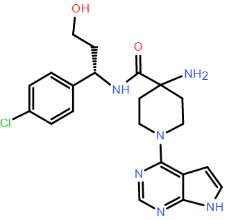 |
| Honokiol | Biphenolic phytochemical;  AKT inhibitor | Antioxidative, anti-inflammatory, antiangiogenic, and anticancer | Honokiol exhibited remarkable inhibition of SARS-CoV-2 replication and SARS-CoV-2 infection(23) | NA | 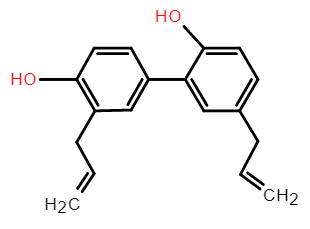 |
| Betrixaban | Non-vitamin K oral anticoagulant | Venous thromboembolism | Potentially repurposable drugs for COVID-19 treatment(24) | DB12364 | 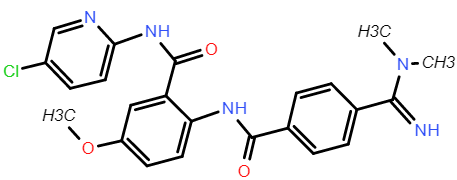 |
| BX-795 | 3-phosphoinositide-dependent kinase 1 (PDK1) inhibitor | Cancers  Anti-HSV activity | Targeting associated pathways in COVID-19(25) | NA | 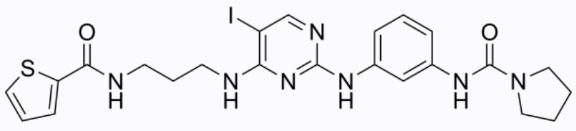 |
| SN-38 | Active metabolite of the [Topoisomerase](https://www.medchemexpress.cn/Targets/Topoisomerase.html) I inhibitor Irinotecan | Cancers | Target SARS-CoV-2 and act on the biological pathways altered in COVID-19(26) | DB05482 | 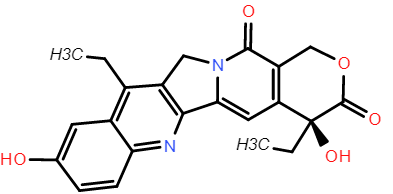 |

**References**

1. Hempel T, Elez K, Kruger N, Raich L, Shrimp JH, Danov O, et al. Synergistic inhibition of SARS-CoV-2 cell entry by otamixaban and covalent protease inhibitors: pre-clinical assessment of pharmacological and molecular properties. Chem Sci. 2021;12(38):12600-9.

2. Shrimp JH, Janiszewski J, Chen CZ, Xu M, Wilson KM, Kales SC, et al. Suite of TMPRSS2 Assays for Screening Drug Repurposing Candidates as Potential Treatments of COVID-19. ACS Infect Dis. 2022;8(6):1191-203.

3. Pohl MO, Busnadiego I, Marrafino F, Wiedmer L, Hunziker A, Fernbach S, et al. Combined computational and cellular screening identifies synergistic inhibition of SARS-CoV-2 by lenvatinib and remdesivir. J Gen Virol. 2021;102(7):001625.

4. Rehman MU, Ali A, Ansar R, Arafah A, Imtiyaz Z, Wani TA, et al. In Silico molecular docking and dynamic analysis of natural compounds against major non-structural proteins of SARS-COV-2. J Biomol Struct Dyn. 2022:1-17.

5. Shah Z, Gohar UF, Jamshed I, Mushtaq A, Mukhtar H, Zia-Ui-Haq M, et al. Podophyllotoxin: History, Recent Advances and Future Prospects. Biomolecules. 2021;11(4):603.

6. Chen L, Guan WJ, Qiu ZE, Xu JB, Bai X, Hou XC, et al. SARS-CoV-2 nucleocapsid protein triggers hyperinflammation via protein-protein interaction-mediated intracellular Cl(-) accumulation in respiratory epithelium. Signal Transduct Target Ther. 2022;7(1):255.

7. Adil MS, Verma A, Rudraraju M, Narayanan SP, Somanath PR. Akt-independent effects of triciribine on ACE2 expression in human lung epithelial cells: Potential benefits in restricting SARS-CoV2 infection. J Cell Physiol. 2021;236(9):6597-606.

8. Pathak Y, Mishra A, Choudhir G, Kumar A, Tripathi V. Rifampicin and Letermovir as potential repurposed drug candidate for COVID-19 treatment: insights from an in-silico study. Pharmacol Rep. 2021;73(3):926-38.

9. Xu Y, Chen K, Pan J, Lei Y, Zhang D, Fang L, et al. Repurposing clinically approved drugs for COVID-19 treatment targeting SARS-CoV-2 papain-like protease. Int J Biol Macromol. 2021;188:137-46.

10. Bellis A, Mauro C, Barbato E, Trimarco B, Morisco C. The Rationale for Angiotensin Receptor Neprilysin Inhibitors in a Multi-Targeted Therapeutic Approach to COVID-19. Int J Mol Sci. 2020;21(22):8612.

11. Xia J, Tang W, Wang J, Lai D, Xu Q, Huang R, et al. SARS-CoV-2 N Protein Induces Acute Lung Injury in Mice via NF-kB Activation. Front Immunol. 2021;12:791753.

12. Ohashi H, Watashi K, Saso W, Shionoya K, Iwanami S, Hirokawa T, et al. Potential anti-COVID-19 agents, cepharanthine and nelfinavir, and their usage for combination treatment. iScience. 2021;24(4):102367.

13. Hijikata A, Shionyu-Mitsuyama C, Nakae S, Shionyu M, Ota M, Kanaya S, et al. Evaluating cepharanthine analogues as natural drugs against SARS-CoV-2. FEBS Open Bio. 2022;12(1):285-94.

14. Yuan S, Chan JFW, Chik KKH, Chan CCY, Tsang JOL, Liang R, et al. Discovery of the FDA-approved drugs bexarotene, cetilistat, diiodohydroxyquinoline, and abiraterone as potential COVID-19 treatments with a robust two-tier screening system. Pharmacol Res. 2020;159:104960.

15. Terali K, Baddal B, Gulcan HO. Prioritizing potential ACE2 inhibitors in the COVID-19 pandemic: Insights from a molecular mechanics-assisted structure-based virtual screening experiment. J Mol Graph Model. 2020;100:107697.

16. Hou Y, Ge S, Li X, Wang C, He H, He L. Testing of the inhibitory effects of loratadine and desloratadine on SARS-CoV-2 spike pseudotyped virus viropexis. Chem Biol Interact. 2021;338:109420.

17. Yadalam PK, Balaji TM, Varadarajan S, Alzahrani KJ, Al-Ghamdi MS, Baeshen HA, et al. Assessing the therapeutic potential of agomelatine, ramelteon, and melatonin against SARS-CoV-2. Saudi J Biol Sci. 2022;29(5):3140-50.

18. Zou Z, Shan H, Sun D, Xia L, Shi Y, Wan J, et al. Parthenolide reveals an allosteric mode to inhibit the deISGylation activity of SARS-CoV‑2 papain-like protease. Acta Biochim Biophys Sin (Shanghai). 2022;54(8):1133-9.

19. Lakhera S, Devlal K, Ghosh A, Chowdhury P, Rana M. Modelling the DFT structural and reactivity study of feverfew and evaluation of its potential antiviral activity against COVID-19 using molecular docking and MD simulations. Chem Zvesti. 2022;76(5):2759-76.

20. Bahrami M, Kamalinejad M, Latifi SA, Seif F, Dadmehr M. Cytokine storm in COVID-19 and parthenolide: Preclinical evidence. Phytother Res. 2020;34(10):2429-30.

21. Coenen S, van der Velden AW, Cianci D, Goossens H, Bongard E, Saville BR, et al. Oseltamivir for coronavirus illness: post-hoc exploratory analysis of an open-label, pragmatic, randomised controlled trial in European primary care from 2016 to 2018. Br J Gen Pract. 2020;70(696):e444-e9.

22. Sun F, Mu C, Kwok HF, Xu J, Wu Y, Liu W, et al. Capivasertib restricts SARS-CoV-2 cellular entry: a potential clinical application for COVID-19. Int J Biol Sci. 2021;17(9):2348-55.

23. Tanikawa T, Hayashi T, Suzuki R, Kitamura M, Inoue Y. Inhibitory effect of honokiol on furin-like activity and SARS-CoV-2 infection. J Tradit Complement Med. 2022;12(1):69-72.

24. Matschinske J, Salgado-Albarran M, Sadegh S, Bongiovanni D, Baumbach J, Blumenthal DB. Individuating Possibly Repurposable Drugs and Drug Targets for COVID-19 Treatment Through Hypothesis-Driven Systems Medicine Using CoVex. Assay Drug Dev Technol. 2020;18(8):348-55.

25. Wang T, Zhao M, Ye P, Wang Q, Zhao Y. Integrated Bioinformatics Analysis for the Screening of Associated Pathways and Therapeutic Drugs in Coronavirus Disease 2019. Arch Med Res. 2021;52(3):304-10.

26. Cava C, Bertoli G, Castiglioni I. Potential drugs against COVID-19 revealed by gene expression profile, molecular docking and molecular dynamic simulation. Future Virol. 2021: 10.2217/fvl-2020-0392.
